# The UBC Domain Is Required for BRUCE to Promote BRIT1/MCPH1 Function in DSB Signaling and Repair Post Formation of BRUCE-USP8-BRIT1 Complex

**DOI:** 10.1371/journal.pone.0144957

**Published:** 2015-12-18

**Authors:** Chunmin Ge, Lixiao Che, Chunying Du

**Affiliations:** Department of Cancer and Cell Biology, University of Cincinnati, College of Medicine, Cincinnati, OH, 45267, United States of America; University of Alabama at Birmingham, UNITED STATES

## Abstract

BRUCE is implicated in the regulation of DNA double-strand break response to preserve genome stability. It acts as a scaffold to tether USP8 and BRIT1, together they form a nuclear BRUCE-USP8-BRIT1 complex, where BRUCE holds K63-ubiquitinated BRIT1 from access to DSB in unstressed cells. Following DSB induction, BRUCE promotes USP8 mediated deubiquitination of BRIT1, a prerequisite for BRIT1 to be released from the complex and recruited to DSB by binding to γ-H2AX. BRUCE contains UBC and BIR domains, but neither is required for the scaffolding function of BRUCE mentioned above. Therefore, it remains to be determined whether they are required for BRUCE in DSB response. Here we show that the UBC domain, not the BIR domain, is required for BRUCE to promote DNA repair at a step post the formation of BRUCE-USP8-BRIT1 complex. Mutation or deletion of the BRUCE UBC domain did not disrupt the BRUCE-USP8-BRIT1 complex, but impaired deubiquitination and consequent recruitment of BRIT1 to DSB. This leads to impaired chromatin relaxation, decreased accumulation of MDC1, NBS1, pATM and RAD51 at DSB, and compromised homologous recombination repair of DNA DSB. These results demonstrate that in addition to the scaffolding function in complex formation, BRUCE has an E3 ligase function to promote BRIT1 deubiquitination by USP8 leading to accumulation of BRIT1 at DNA double-strand break. These data support a crucial role for BRUCE UBC activity in the early stage of DSB response.

## Introduction

DNA double-strand breaks (DSBs) are recognized as the most toxic DNA lesions. Failure in the repair of DSB can induce genome instability, an event implicated in a number of human diseases including cancers, neurodegeneration, and aging [1–3]. It is not surprising that there exist cellular DNA damage response (DDR) pathways to detect, signal and repair DNA damage to counteract the impact of DSB and preserve genome stability. To accomplish DNA repair, it often requires protein-protein interactions and formation of large protein complexes to transduce and amplify the damage signals. A large body of research indicate that formation of many of these protein complexes depends on post-translational modifications, including but not limited to phosphorylation, ubiquitination, and sumoylation to remodel the chromatin regions flanking damaged DNA [4,5]. Among which, ubiquitination by the covalent attachment of the 76 amino acid ubiquitin protein (Ub) to protein substrates, plays critical roles not only for targeting the modified protein for proteasomal degradation, but also for them to gain new functions, change subcellular localization and alter interacting partners. Ubiquitination of histones at DNA DSBs facilitates the recruitment of downstream repair proteins. A lot of insight into how ubiquitin signaling regulates DNA DSB response is provided by the studies of the two E3 ubiquitin ligases RNF8 and RNF168 in the modification of histone H2A and H2AX flanking DSB. In response to DSB induction, RNF8 is recruited to damaged chromatin by binding to phosphorylated MDC1 which is phosphorylated mainly by the DNA damage kinase ATM. At DSB, RNF8 plays a critical role in the ubiquitination of H2A type of histones [6,7]. It seems to be critical for initiation of the ubiquitination modification of H2A type of histones, whereas RNF168, recruited to DSB site by recognition of RNF8 ubiquitinated products, catalyzes the bulk histone modifications flanking DSB at Lys-13 and Lys-15 of H2A and H2AX [8–11]. These histone ubiquitinated products with K63 or K27 Ub linkage create the docking sites for the recruitment of the repair proteins 53BP1 and BRCA1 at DSB for repair [6,7,9,10,12]. In addition to DNA DSB repair, ubiquitination also plays an essential role in the repair of DNA inter strand cross-links by the Fanconi anemia (FA) pathway [13]. At the center of this pathway is the mono-ubiquitination of the FANCD2 by the multisubunit FA core complex in which FANCL is the catalytic E3 ubiquitin ligase. The mono-ubiquitination is required for targeting FANCD2 to damaged chromatin and ubiquitinated FANCD2 is a platform for the recruitment of additional proteins that coordinate efficient homologous recombination repair of damaged DNA [14–17].

Deubiquitination, the reverse process of ubiquitination catalyzed by deubiquitinating enzymes (Dubs), is equally important for the regulation of DNA damage signaling and repair [18]. One multidimentional screening approach has identified Dubs that function in DNA damage checkpoint and genome stability maintenance [19]. Alternative approaches of candidate Dub analysis have identified several Dubs that specifically counteract RNF8 and RNF168-mediated DNA DSB-induced ubiquitination of histones through removal of ubiquitin moiety from Ub-H2A and Ub-H2AX [20–22]. USP3, USP44, and USP16 are identified to counteract the function of RNF168 by promoting deubiquitination of H2A and H2AX [20,21]. As a result, they negatively regulate DSB response [20–22]. Moreover, the pioneer work a decade ago in FA studies has identified the Dub USP1 as a novel component of the FA pathway promotes deubiquitination of FANCD2 for the repair of interstrand cross-linked DNA [23]. Removal of ubiquitin from FANCD2 induces dissociation of FANCD2 from damaged chromatin, allowing the next round of ubiquitination to occur [17].

In contrast to the substantial understanding for ubiquitination in targeting repair factors to damage sites and that deubiquitination for opposing the process, little is known about deubiquitination in targeting repair factors to damaged chromatin. Our recent study has revealed such a cellular process that deubiquitination of K63-Ub-BRIT1 at the region of aa 566–655 mediated by the Dub USP8 is needed for targeting BRIT1 to site of damaged chromatin [24]. In this scenario, BRUCE, a large protein with molecular mass of 528 kDa [25,26] promotes the BRIT1-SWI-SNF pathway of DSB response for DNA repair and genome stability [24]. Once BRIT1 is recruited to DSB-flanking chromatin, it recruits the chromatin remodeler SWI-SNF complex to the site and therefore the chromatin structures is altered to a relaxed configuration for DNA repair. Like many repair and signaling proteins, BRIT1 is recruited to site of DSB by binding to γ-H2AX at DSB-flanking chromatin. The fate of BRIT1 being away from DSB or recruited to DSB by γ-H2AX is regulated by BRUCE and USP8. In resting cells, BRUCE acts as an scaffold to tether USP8 and BRIT1 (K63-ubiquitinated) onto an assembly platform, forming a nuclear complex BRUCE-USP8-BRIT1 and as such, ubiquitinated BRIT1 is sequestered onto the complex [24]. Following DNA damage induction, BRUCE and USP8 work together to remove the Ub chains to unleash BRIT1 for its recruitment to DSB by binding to γ-H2AX and promotes homologous recombination (HR) repair of DNA DSB and preserve genome stability. Depletion of BRUCE in cells impairs accumulation of key repair proteins to site of damaged chromatin, results in elevated levels of DNA DSB, and several forms of chromosomal abnormalities, including DNA breaks, gaps and end-to-end association. While BRUCE and USP8 do not actually localize to site of DNA damage or repair DNA themselves, they regulate the deubiquitination of BRIT1, paving the way for BRIT1 to enter the scene and fix broken strands [24].

BRUCE protein contains two well-characterized domains. One is the N-terminal Baculoviral IAP Repeat (BIR), a signature domain present in all inhibitor of apoptosis proteins (IAPs). The other is the C-terminal Ubiquitin conjugating (UBC), the E2 in the three sequential enzymes for protein ubiquitination [26–28]. The BIR domain makes BRUCE an IAP and when overexpressed, BRUCE uses its BIR to suppress apoptosis and antagonize pro-apoptotic function of Smac/Diablo and caspase-3, -6, -7, -9 [26,29–33]. In contrast to BIR domain, the biological significance of BRUCE UBC remains elusive. Biochemical studies have indicated that the UBC domain of BRUCE has both Ub conjugase (E2) and Ub-protein ligase (E3) functions, making BRUCE a hybrid E2/E3 enzyme [29]. Although BRUCE UBC is implicated in the ubiquitination of the mitochondrial proapoptotic protein Smac [27], the biological function of this modification remains unknown.

In the recently elucidated role of BRUCE in the regulation of DNA damage signaling and repair, neither the BIR or UBC is required for the scaffold function of BRUCE in the formation of BRUCE-USP8-BRIT1 complex [24]. Therefore it remains as an interesting question as for whether these domains are involved in the BRUCE-USP8-BRIT1 pathway for DSB signaling and repair. This question is addressed in this study. Our results showed that the UBC, not the BIR domain, plays a critical role in DNA repair. Catalytic inactivation of UBC domain impairs BRIT1 foci formation, chromatin relaxation and homologous recombination repair. Further, the function of BRUCE in DNA repair is independent of its role in anti-apoptosis and pro-cytokinesis.

## Materials and Methods

### Cell culture and transfection

Human U2OS and HEK 293T cell lines were purchased from ATCC and cultured in DMEM high glucose medium with 10% fetal bovine serum and 1% penicillin/streptomycin at 37°C in a CO_2_ (5%) incubator. Plasmid or siRNA transfection was mediated by Lipofectamine 2000 or Lipofectamine RNAiMAX (Invitrogen), respectively, following manufacturer’s instruction.

### Antibodies

Antibodies against BRUCE from Calbiochem (#AP1031) and Bethyl (#A300-367A); BRIT1 from Cell Signaling (#4120) and Abcam (#ab2612 and #ab121277); α-Tubulin from Sigma (#T9026); FLAG (M2) from Sigma (#A8592 and #F3165); c-Myc (#sc-40 and #sc-789) and GFP (#sc-9996) from Santa Cruz; pATM from BD Biosciences (#DR1002); MDC1 from Novus (#NB100-395); NBS1 (#GTX30125), Rad51 (#GTX100469) and γ-Tubulin (#GTX113286)from GeneTex.

### Reagents and siRNAs

Micrococcal nuclease from Sigma (#N3755); BRUCE siRNAs and one control siRNA were synthesized by Dharmacon. BRUCE siRNA#1, #2, and #3 sequences are GGUACAAUCACAUCUAGCAdTdT, GACCUUAAUGGAAUCUUGUdTdT, and GUUAUGAGCUGCUUGUAGAdTdT, respectively. Control siRNA sequence is UUCUCCGAACGUGUCACGUdTdT [34].

### Preparation of chromatin-containing whole cell lysates

Cell pellets were lysed and sonicated to elute whole cell lysate plus chromatin in NETN buffer (50 mM Tris-HCI pH 8.0, 150 mM NaCl, 1 mM EDTA, 0.5% NP40) with protease inhibitor tablets (Roche) and phosphatase inhibitors of 10 mM NaF and 50 mM β-glycerophosphate [35]. The lysates were centrifuged at 15,000 g for 20 minutes and the supernatant was collected as cell-free extracts.

### Immunoblotting

Protein extracts (40–100 μg) were resolved by SDS-PAGE and transferred to nitrocellulose filter. The filter was blocked with 5% dry milk in PBST for 1 hour at room temperature, followed by incubation with primary antibody overnight at 4°C or 2 hours at room temperature. The filter was then washed in PBST 5 times for 5 minutes each, followed by incubation with HRP-conjugated secondary antibody for 1 hour at room temperature. After washing with PBST, the filter was developed with ECL for 1 minute and exposed to X-ray film.

### Immunofluorescent staining of DNA damage induced nuclear foci

Cells cultured in 8-well chamber slide or on coverslips in 6-well culture plates were irradiated with a Faxitron X-ray system (RX-650). For staining of pATM and MDC1 foci, cells were fixed in 4% paraformaldehyde (PFA) on ice for 15 minutes, then permeabilized in 0.5% Triton X-100 at room temperature for 12 minutes. For staining Rad51 foci, cells were pre-extracted for 6 minutes on ice in extraction buffer (10 mM PIPES pH 6.8, 300 mM sucrose, 20 mM NaCl, 3 mM MgCl_2_, 0.5% Triton X-100), and then fixed with 4% PFA on ice for 15 minutes. For staining of NBS1 and BRIT1 foci or co-immunostaining of BRIT1 with FLAG-BRUCE or FLAG-BRUCE-N/-C, cells were fixed in methanol at -20°C for 20 minutes. After blocking with 3% BSA, samples were incubated with primary antibodies overnight on a shaker in a cold room or 2 hours at room temperature followed by incubation with secondary antibodies conjugated with AlexaFluro 488 or AlexaFluro 594 for 1 hour at room temperature. After washes, samples were examined under a Zeiss LSM 710 confocal microscope.

### Cytokinesis assessment

Cells were fixed with methanol at -20°C for 20 min, followed by centrosome staining with γ-tubulin antibody or combination with pATM/BRIT1 antibody and cell nucleus counterstained with DAPI. The number of centrosome and nucleus in each cell was counted under fluorescence microscope.

### U2OS Clone #16 with stable expression of DOX-inducible shBRUCE

siRNA targeting BRUCE (GGCACAGCAGCTCTTATCA) was used to generate the shRNA: 5’-atcccGGCACAGCAGCTCTTATCAttcaagagaTGATAAGAGCTGCTGTGCCttttta-3’ and 5’-tcgaatttttCCGTGTCGTCGAGAATAGTaagttctctACTATTCTCGACGACACGGcc-3’. Eight tandem repeats of each shRNA, with an H1 promoter, were cloned into pSUPERIOR.puro vector following the manufacturer’s instruction (Oligoengine). The construct was then transfected with Lipofectamine 2000 into a U2OS cell line in which a Tet repressor (TetR) was stably expressed. The transfected cells were selected for clones that integrated with both the shRNA construct and TetR by puromycin (1 μg/ml) and blasticidin (5 μg/ml), respectively. More than six positive stable clones were identified by immunoblotting in which DOX (1 μg/ml) treatment for 4–6 days ablated BRUCE expression, and clone #16 was used in this study and results confirmed in other clones.

### Reconstitution of shRNA-resistant full-length cDNA expressing BRUCE in U2OS Clone #16

A pCI-Neo mammalian expression vector (Promega) containing cDNA encoding the full-length BRUCE protein consisting 4857 amino acids (pCI-Neo-FLAG-BRUCE) was made from piecing a cDNA encoding a BRUCE isoform consisting of 4829 amino acids together with a short N-terminal fragment encoding 28 amino acids with a FLAG tag engineered to the N-terminus of the construct. Mutations in the cDNA were corrected and verified by DNA sequencing. Scramble/wobble mutations shown in italic AGCCCAACAACTGTTGTCC were introduced to this BRUCE construct to make it resistant to shRNA. BIR mutation/deletion and UBC mutation/deletion were introduced to full length BRUCE by site-directed mutagenesis kit. Five pCI-Neo-FLAG-BRUCE variant constructs were each transfected into U2OS clone #16 by Lipofectamine 2000. About 200 cell clones resistant to puromycin (1 μg/ml) and G418 (1 mg/ml) were obtained and ~20 of them were verified for stable expression of full-length BRUCE by immunoblotting and for their expression level to be the same or similar to endogenous BRUCE, two set of these variant BRUCE stable clones were used in the study.

### Truncated constructs

Full-length human BRUCE cDNA in pCI-Neo vector (24) was digested by restriction endonucleases *Sal*l (cutting pCI-Neo) and *SnaB*I (cutting BRUCE cDNA) to generate the N-terminal fragment encoding 1–2025 amino acid residues, or by *SnaB*I and *Not*I (cutting pCI-Neo) to generate the C-terminal fragment encoding 2024–4857 amino acid residues. The resulting fragments were subcloned into pCI-Neo expression vector with FLAG tag fused to the N-terminus of the fragment.

### Ubiquitination assay

Ubiquitination assay was conducted in the chromatin enriched fraction. Cells were lysed and fractioned following the protocol previously described with minor modification [36]. Briefly, U2OS cells were lysed in buffer I (50 mM HEPES pH 7.5, 150 mM NaCl, 1 mM EDTA, 0.05% NP40, and protease and phosphatase inhibitors) for 5 minutes on ice. After centrifugation at 3500 rpm for 5 minutes at 4°C, supernatant was removed and the precipitate was washed once with buffer I, and then extracted with buffer II (50mM Tris-Cl pH 7.5, 150 mM NaCl, 1% NP40, 0.5% deoxycholate, 0.1% SDS, protease and phosphatase inhibitors) plus sonication. The extract was collected after centrifugation and subjected to c-Myc immunoprecipitation as previously described (24).

### Reverse transcription polymerase chain reaction (RT-PCR) assay

mRNAs were prepared with Trizol reagent (Invitrogen) following the manufacturer’s instructions. Reverse transcription was carried out with SuperScript II Reverse Transcriptase (Invitrogen) following the manufacturer’s instructions. Primers for amplification of endogenous C-BRUCEDNA are TTACTATAGAGTGAGGGGTTG (forward) and TCCTCTCTCCTCTACAGAGCCTC (reverse). Amplification of exogenous BRUCE was carried out by using the same forward primer for amplification of endogenous BRUCE and with a different reverse primer TAA CCCTCACTAAAGGGAAGCGG.

### Micrococcal nuclease digestion assay

Micrococcal nuclease digestion was performed according to the protocol from Dr. Rachid Drissi at Cincinnati Children’s Hospital Medical Center. Briefly, cells cultured in 6-cm plates were treated with 5 Gy of IR; after 1 hour culture at 37°C, media was removed and cells were treated with 1 ml of 0.01% egg lysolecithin in PS1 buffer (150 mM sucrose, 80 mM KCl, 35 mM HEPES pH 7.4, 5 mM K_2_PO_4_, 5 mM MgCl_2_ and 0.5 mM CaCl_2_) for 90 seconds. After removal of PS1, cells were incubated with 0.8 ml of PS2 buffer (20 mM sucrose, 50 mM Tris-Cl pH 7.5, 50 mM NaCl and 2 mM CaCl_2_) containing 2 U/ml micrococcal nuclease at room temperature for the desired time (2–5 minutes). After digestion, PS2 was removed and samples were treated with 1 ml of permeabilization stop solution (20 mM Tris-Cl pH 8.0, 20 mM NaCl, 20 mM EDTA and 1% SDS) containing 0.6 mg/ml proteinase K, followed by the addition of 0.5 ml of lysis dilution buffer (150 mM NaCl and 5 mM EDTA) and transfer of the solution to a 15 ml polypropylene tube for incubation at 37°C overnight. TE buffer was then added to the tube to reach 3 ml in total. Genomic DNA was purified with phenol-chloroform extraction and was separated by electrophoresis in a 1% agarose gel.

### Homologous recombination (HR) assay

The HR assay was performed in U2OS-DR-GFP cell lines [37,38]. Cells cultured in 6 well plates at 40% confluency were transfected twice with control or target siRNA. 24 hours after the second round of siRNA transfection, cells were transfected with I-*Sce*I-expressing pCBASce plasmid. After an additional 48 hours of culture, cells were harvested and GFP positive cells were analyzed by flow cytometry (BD LSRII analyser).

## Results

### The N-terminal or C-terminal half of BRUCE is not sufficient to support formation of BRIT1 repair foci

**Fig 1:**
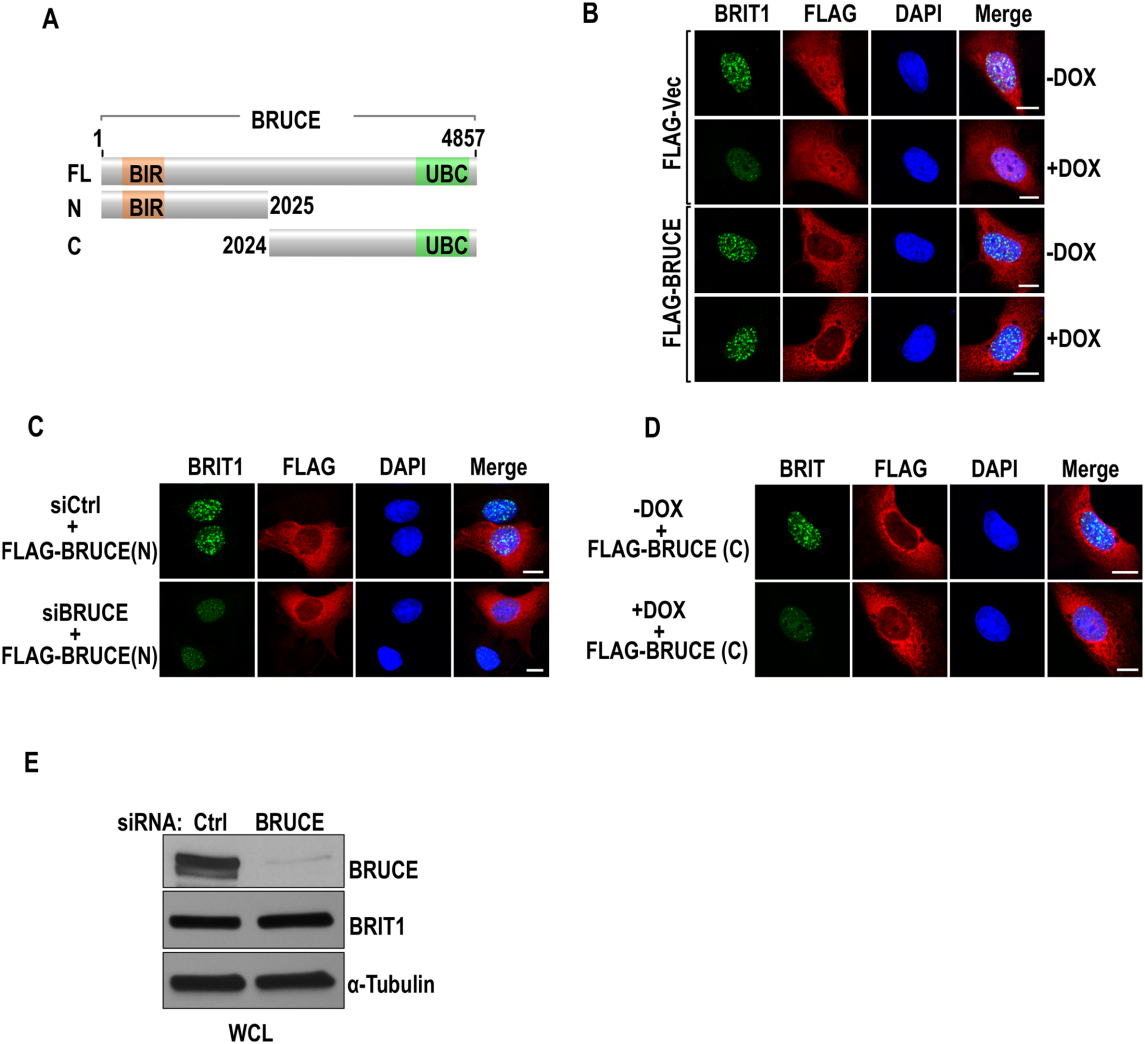
Truncated N-BRUCE or C-BRUCE cannot support BRIT1 foci formation in irradiated U2OS cells. (**A**) Diagram depicting plasmid constructs expressing human full length (FL) BRUCE (4857 amino acids), N-BRUCE (aa 1–2025), and C-BRUCE (aa 2024–4857) with BIR and UBC domains indicated. (**B**) BRUCE restores formation of IR-induced BRIT1 foci. DOX-inducible shBRUCE-stable-expression U2OS cells (U2OS-shBRUCE, [24]) were transiently transfected with a FLAG vector or a construct expressing FLAG fused with BRUCE (FLAG-BRUCE). Cells were treated with DOX to induce expression of shBRUCE and followed by exposure to IR (5 Gy). 1 hr post IR, cells were immunofluorescence stained with antibodies specific for FLAG (red) and endogenous BRIT1 (green) with cell nucleus counterstained with DAPI (blue). Bars, 10 μm. (**C**) N-BRUCE cannot support BRIT1 foci formation. U2OS cells with endogenous BRUCE depleted by siBRUCE were transiently transfected with an expression construct of FLAG fused with N-BRUCE (aa 1–2025, depicted in Fig 1A). At 1 hr post IR (5 Gy), cells were immunofluorescence stained with antibodies against FLAG (red) and endogenous BRIT1 (green) with cell nucleus counterstained with DAPI (blue). Bars, 10 μm. (**D**) C-BRUCE cannot support BRIT1 foci formation. DOX-inducible shBRUCE-U2OS cells described in (B) was transiently transfected with a construct expressing FLAG fused C-BRUCE (aa 2024–4857, depicted in Fig 1A). Cells were treated with DOX to induce expression of shBRUCE followed by exposure to IR (5 Gy). 1 hr post exposure, cells were immunofluorescence stained with antibodies against FLAG (red) and endogenous BRIT1 (green) with cell nucleus counterstained with DAPI (blue). Bars, 10 μm. (**E**) BRIT1 protein levels remain unchanged post BRUCE knockdown. BRUCE expression was depleted by siRNA as indicated. Whole cell lysates (WCL) were examined by immunoblotting for BRUCE and BRIT1; α-tubulin blotting as loading control.

Our recent study demonstrated the sequential steps for BRUCE-USP8-BRIT1 to transduce DNA damage signals for DNA repair. The formation of the BRUCE-USP8-BRIT1 complex in unstimulated cells is mediated by N-terminal half of BRUCE (N-BRUCE). This is followed by release of BRIT1 from the complex mediated by deubiquitination of BRIT1 in irradiated cells, and consequently recruitment of deubiquitinated BRIT1 to damaged chromatin by binding to γ-H2AX, forming BRIT1 repair foci that can be observed by BRIT1 immunofluorescence staining of the cell. Although N-BRUCE is sufficient for BRUCE to acts as the scaffold tethering USP8 and BRIT1 onto the complex, it remains unknown whether N-BRUCE is sufficient for the formation of BRIT1 repair foci. In addition, whether the C-terminal half of BRUCE (C-BRUCE) also contributes to the function of the complex remains unclear. To examine this notion, expression constructs of full-length BRUCE, N-BRUCE, and C-BRUCE (Fig 1A, all FLAG-tagged and siRNA resistant) were each transfected into human osteosarcoma U2OS cells pre-depleted of endogenous BRUCE by RNAi. Their ability to restore the formation of BRIT1 DNA repair foci was compared at 1 hr post exposure to IR (5 Gy) by immunofluorescence staining. In contrast to the control that wild type BRUCE rescued the formation of BRIT1 nuclear foci (Fig 1B), N-BRUCE or C-BRUCE could not (Fig 1C and 1D, respectively). The lack of BRIT1 foci is not due to the reduction of BRIT1 expression as the endogenous levels of BRIT1 protein remained unchanged post BRUCE knockdown (Fig 1E). Together, these results indicate that subsequent to the formation of the BRUCE-USP8-BRIT1 complex mediated by N-BRUCE, the C-BRUCE is required for targeting BRIT1 to DSB sites. Since the UBC domain is a major domain present in C-BRUCE, we speculated that the UBC is engaged in the regulation of BRIT1 function in DDR. In addition, despite that BIR is not required for the complex formation [24], it remains an open question as for whether it is needed together with C-BRUCE for targeting BRIT1 to damaged chromatin. Therefore, we investigated the involvement of UBC and BIR domains in BRUCE-regulated BRIT1 DDR function.

### Establishment of stable U2OS cell lines expressing mutant BRUCE variants

**Fig 2:**
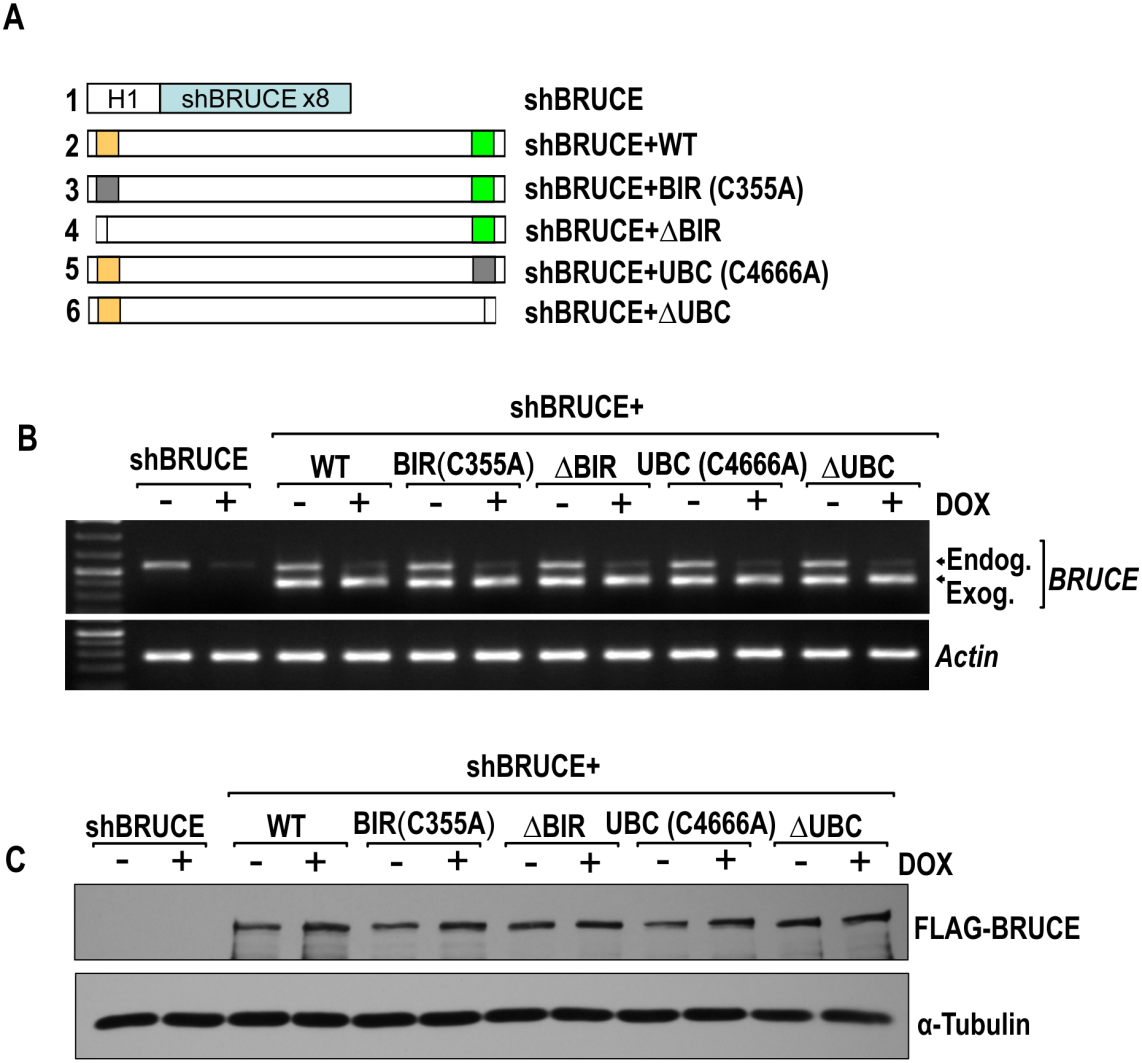
shBRUCE-U2OS cell lines with expression of exogenous BRUCE variants. (**A**) Diagram of a DOX-inducible shBRUCE construct under H1 promoter (tandem eight repeats of shBRUCE sequences, top) and five BRUCE constructs expressing wild type BRUCE, mutant BRUCE with BIR or UBC domain mutated at the active site as indicated by the respective number of amino acid residue, or with the entire domain deleted (Δ). These BRUCE constructs are resistant to shBRUCE by introducing wobble mutation; see Methods for details. (**B**) U2OS cells with stable expression of the H1-shBRUCE construct (U2OS-shBRUCE) were transfected with each of the BRUCE constructs in (A). The resultant samples were selected for stable cell lines expressing each BRUCE variant as indicated on the top. These stable cell lines were left untreated or treated with DOX for 4 days to knockdown BRUCE. RT-PCR analysis of each cell line showing levels of endogenous BRUCE mRNA were significantly decreased after DOX treatment (upper arrow), whereas that of exogenous BRUCE mRNA remained the same across the cell lines (lower arrow); *Actin* as loading control. (**C**) Cell lysates of the above cell lines were immunoblotted with FLAG antibody to examine expression of exogenous BRUCE variants; α-Tubulin blotting as loading control.

The parental U2OS cell line with stable expression of shBRUCE under doxycycline (DOX) induction (U2OS-shBRUCE) and the isogenic line with stable expression of FLAG-tagged, shBRUCE-resistant wild type BRUCE (shBRUCE+WT) were reported previously [24] and they are used as controls in this study. Four new isogenic cell lines were derived from the parental U2OS-shBRUCE cells by stable expression of FLAG-tagged BIR mutant at C355A, BIR deleted (ΔBIR; aa 287–363), UBC mutant at C4666A, or UBC deleted (ΔUBC; aa 4605–4690) (Fig 2A), among which the point mutations inactivate the function of BIR and UBC of BRUCE [29,30]. The resulting cell lines were validated for the knockdown of endogenous BRUCE mRNA and reconstituted expression of exogenous BRUCE mRNA by RT-PCR (Fig 2B, upper and lower bands; respectively; see Methods for details). Furthermore, the expression of FLAG-BRUCE proteins was confirmed by immunoblotting with a FLAG antibody (Fig 2C).

### The UBC but not the BIR domain is required for BRUCE to promote BRIT1 function in DDR

**Fig 3:**
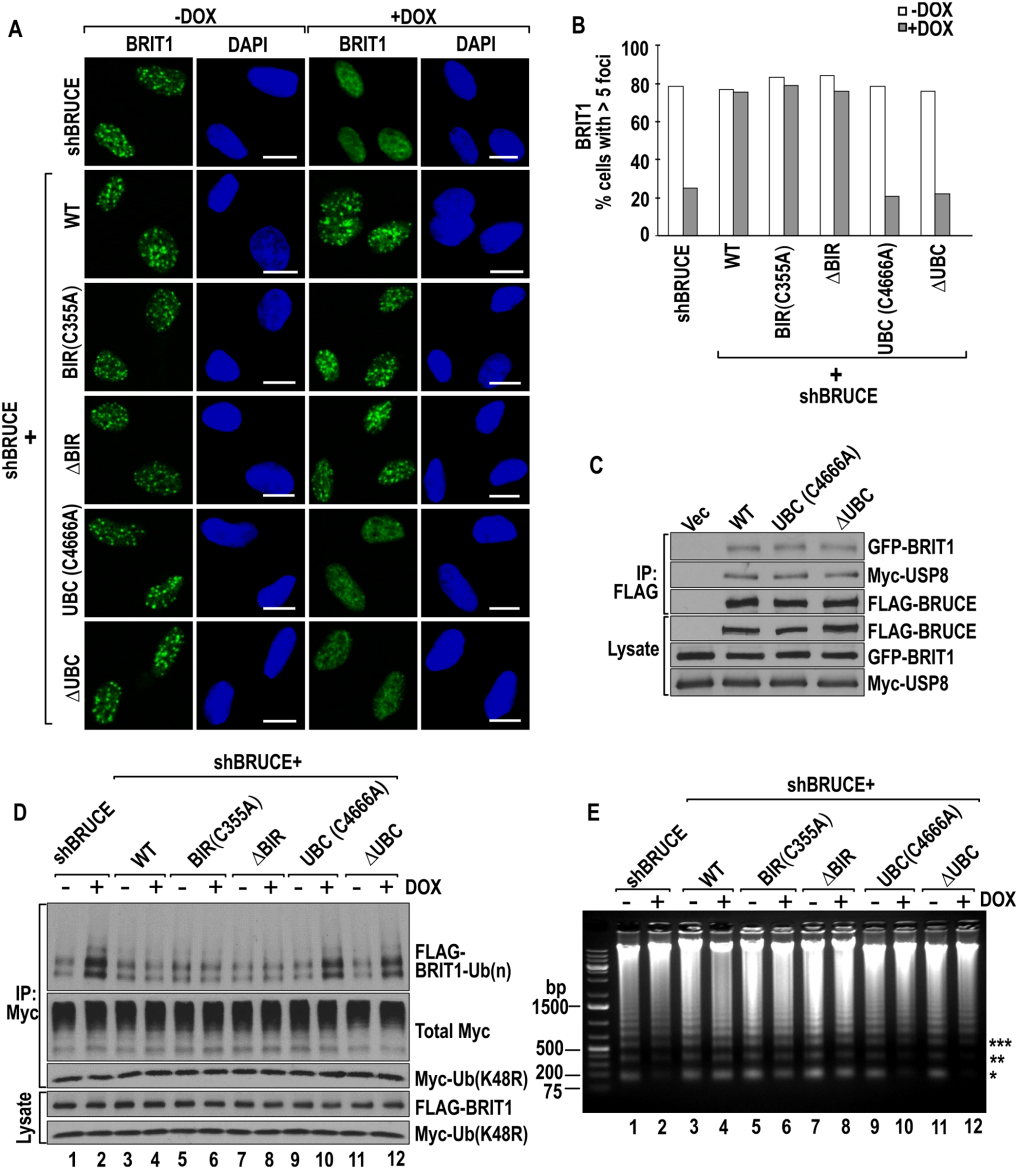
BRUCE UBC domain is required for BRIT1 deubiquitination and its repair foci formation post the formation of the BRUCE-USP8-BRIT1 nuclear complex. (**A**) BRUCE UBC domain is needed for BRIT1 foci formation. U2OS-shBRUCE cells expressing BRUCE variants as indicated to the left were treated with DOX to knockdown endogenous BRUCE. Cells were irradiated (5 Gy), fixed and immunofluorescence stained with BRIT1 antibody. Bars, 10 μm. (**B**) Quantification of the BRIT1 foci results in (A). Cells with positive BRIT1 foci (more than five nuclear foci) were quantitated and a total of more than two hundred cells were counted for each sample. (**C**) UBC mutation or deletion does not affect the binding of BRUCE with USP8 and BRIT1. U2OS-shBRUCE cell lines with stable expression of WT, UBC C4666A mutant, or ΔUBC BRUCE (all FLAG tagged) as indicated on to top were co-transfected with GFP-BRIT1 and Myc-USP8 constructs. BRUCE was isolated from the cell lysates by IP of FLAG followed by immunoblotting of the IP complex with antibodies indicated to the right. (**D**) UBC domain is required for BRIT1 deubiquitination. U2OS-shBRUCE cells were untreated or treated with DOX followed by transfection with GFP-BRIT1 and My-ubiquitin (K48R). After irradiation (5 Gy), cells were subject to immunoprecipitation with anti-Myc antibody to pulldown total ubiquitinated proteins, among which ubiquitinated BRIT1 products were detected by immunoblotting with an anti-GFP antibody. (**E**) UBC domain is needed for chromatin relaxation induced by DNA damage. U2OS-shBRUCE cells with expression of exogenous BRUCE and BRUCE variants as indicated to the top were treated with DOX to knock down endogenous BRUCE. After irradiation (5 Gy), cells were subject to micrococcal nuclease digestion assay. Chromatin relaxation was monitored by the release of nucleosomes. Mono- (*), di- (**) and tri-nucleosomes (***) are indicated.

To investigate the involvement of UBC and BIR domains in BRUCE-regulated BRIT1 DDR function, first we examined whether they are required for BRUCE to promote the formation of BRIT1 repair foci. The above-mentioned six stable U2OS cell lines were treated with DOX to knockdown the expression of endogenous BRUCE. Subsequently they were exposed to IR (5 Gy) and the formation of BRIT1 foci was examined by 1 hr post IR with immunofluorescence staining. Similar to expression of wild type BRUCE, expression of BIR C355A or ΔBIR mutant BRUCE enabled the formation of BRIT1 DNA damage foci (Fig 3A and 3B). However, expression of UBC C4666A or ΔUBC mutant BRUCE resulted in lack of BRIT1 foci formation (Fig 3A and 3B). These results indicate that the UBC but not the BIR domain is required for BRUCE to target BRIT1 to the site of DSB. Given the fact that the integrity of the BRUCE-USP8-BRIT1 complex is essential for targeting BRIT1 to DSB, whether the observed UBC requirement is attributable to its contribution to maintaining the complex integrity was examined. Expression constructs of Myc-USP8 and GFP-BRIT1 were co-transfected into U2OS cells stable expressing FLAG-tagged WT, UBC C4666A or ΔUBC BRUCE. FLAG fused BRUCE in the cell lysates was isolated by immunoprecipitation (IP) with an anti-FLAG M2 antibody. The integrity of the complex was examined for the presence of USP8 and BRIT1 in BRUCE-IP complex by immunoblotting. The results showed that both USP8 and BRIT1 were still co-immunoprecipitated with UBC C355A and ΔUBC mutant BRUCE (Fig 3C), demonstrating that inactivation of BRUCE UBC does not disrupt the integrity of BRUCE-USP8-BRIT1 complex. Thus, these results indicate that the role of UBC domain in enabling BRIT1 foci formation lies at a step post complex formation and prior to the recruitment of BRIT1 to DSB.

Deubiquitination of BRIT1 by BRUCE-dependent USP8 is an intermediate step between the complex formation and BRIT1 recruitment to DSB. It is also a prerequisite for BRIT1 recruitment [24]. Next, whether the UBC domain of BRUCE is implicated in this step was examined. The six BRUCE U2OS cell lines were treated with DOX to deplete the expression of endogenous BRUCE followed by transfection with FLAG-BRIT1 and Myc-Ub (48R) expression constructs. After exposure to IR, the total ubiquitinated proteins were isolated from the cell extracts by IP with anti-Myc antibody. In the IP products the levels of ubiquitinated BRIT1 products were assessed by BRIT1 immunoblotting and the levels of ubiquitinated BRIT1 was then compared. Same as reported previously that BRUCE promotes BRIT1 deubiquitination [24] which is shown as control in Fig 3D (lanes 1–4), BIR C355A and ΔBIR mutant BRUCE also promoted BRIT1 deubiquitination (Fig 3D, lanes 5–8). In contrast, UBC C4666A and ΔUBC mutant BRUCE could not promote BRIT1 deubiquitination and ended up with elevated levels of BRIT1 ubiquitinated products (Fig 3D, lanes 9–12). Together these results demonstrated that the UBC but not the BIR domain is required for BRUCE to promote USP8 deubiquitination of BRIT1 in response to IR exposure.

Once deubiquitinated, BRIT1 is released from the BRUCE-USP8-BRIT1 complex and targeted to DSBs by binding to γ-H2AX at sites of DSB [24], where BRIT1 recruits the SWI/SNF chromatin remodeling complex to facilitate relaxation of chromatin configuration for the access of repair factors to damaged chromatin [39]. Therefore, we further examined if the UBC domain of BRUCE is critical for chromatin relaxation by micrococcal nuclease digestion assay. Compared to compact chromatin, relaxed chromatin configuration is more accessible by micrococcal nuclease digestion and, as a result, more nucleosomes are released. The results showed that same as the wild type BRUCE, BIR-inactivated BRUCE restored the relaxed chromatin configuration induced by depletion of endogenous BRUCE (Fig 3E, lanes 1–8). However, BRUCE with UBC inactivated resulted in decreased release of nucleosomes (Fig 3E, lanes 9–12), indicating that UBC-inactivated BRUCE could not restore the chromatin structure to a relaxed configuration. Together, these results demonstrated that it is the UBC domain but not the BIR that is required for BRUCE to promote chromatin relaxation through facilitating BRIT1-SWI-SNF pathway for chromatin relaxation at damaged chromatin. The dispensability of BRUCE BIR for deubiquitination of BRIT1, localization of BRIT1 to DSB, and promotion of chromatin relaxation supports a notion that the DDR function of BRUCE is separated from its anti-apoptotic function.

### BRUCE UBC domain is required for the accumulation of early DDR signaling proteins to sites of DSB

**Fig 4:**
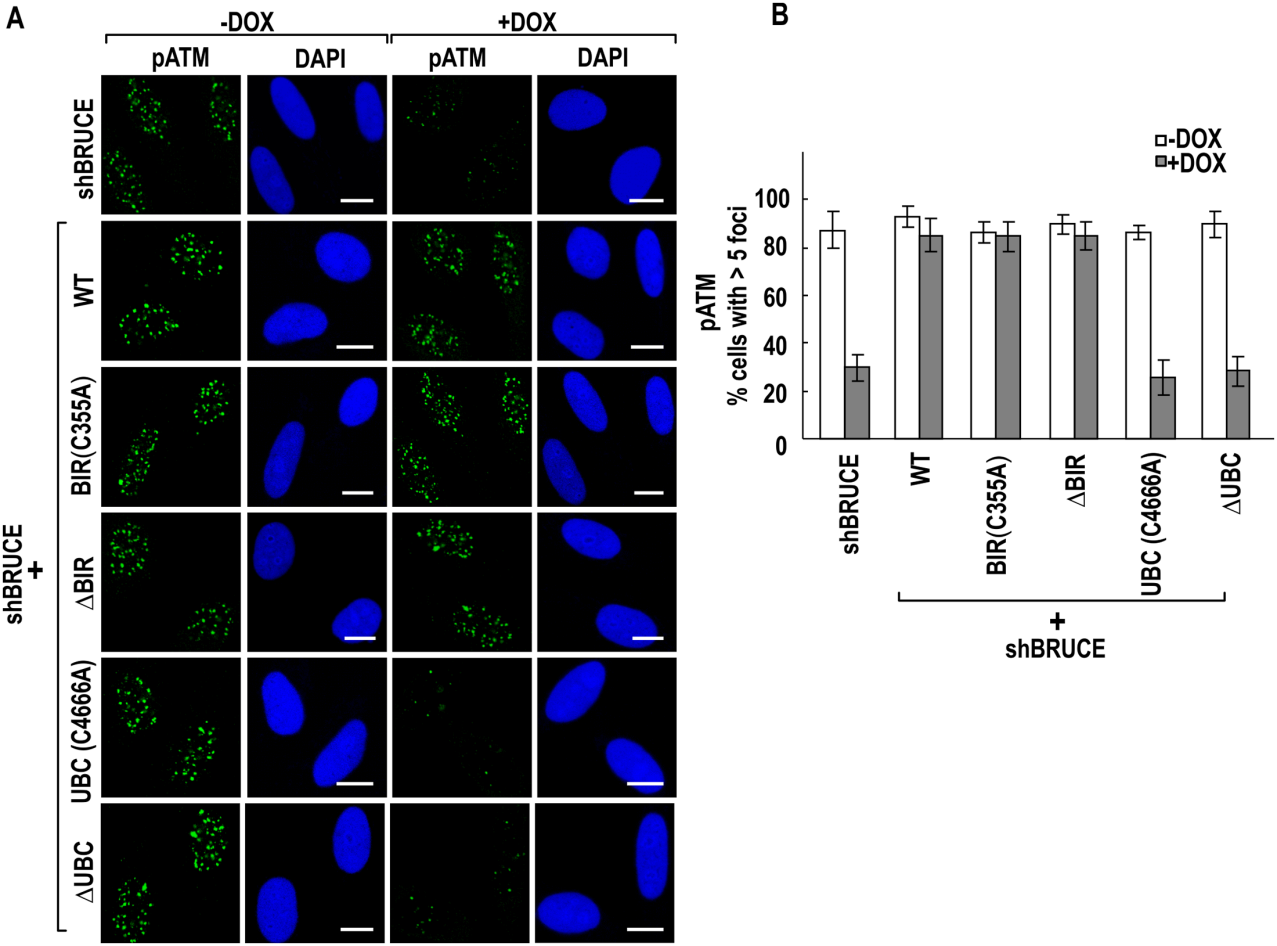
BRUCE UBC domain is required for pATM repair foci formation. U2OS-shBRUCE cells with stable expression of BRUCE or BRUCE mutants as indicated to the left were treated with DOX to knockdown endogenous BRUCE. After irradiation, cells were fixed and immunofluorescence stained for pATM (**A**) and quantified for cells with more than five nuclear foci (**B**). Bars, 10 μm. Error bars represent standard deviation from a triplicate of a representative experiment.

**Fig 5:**
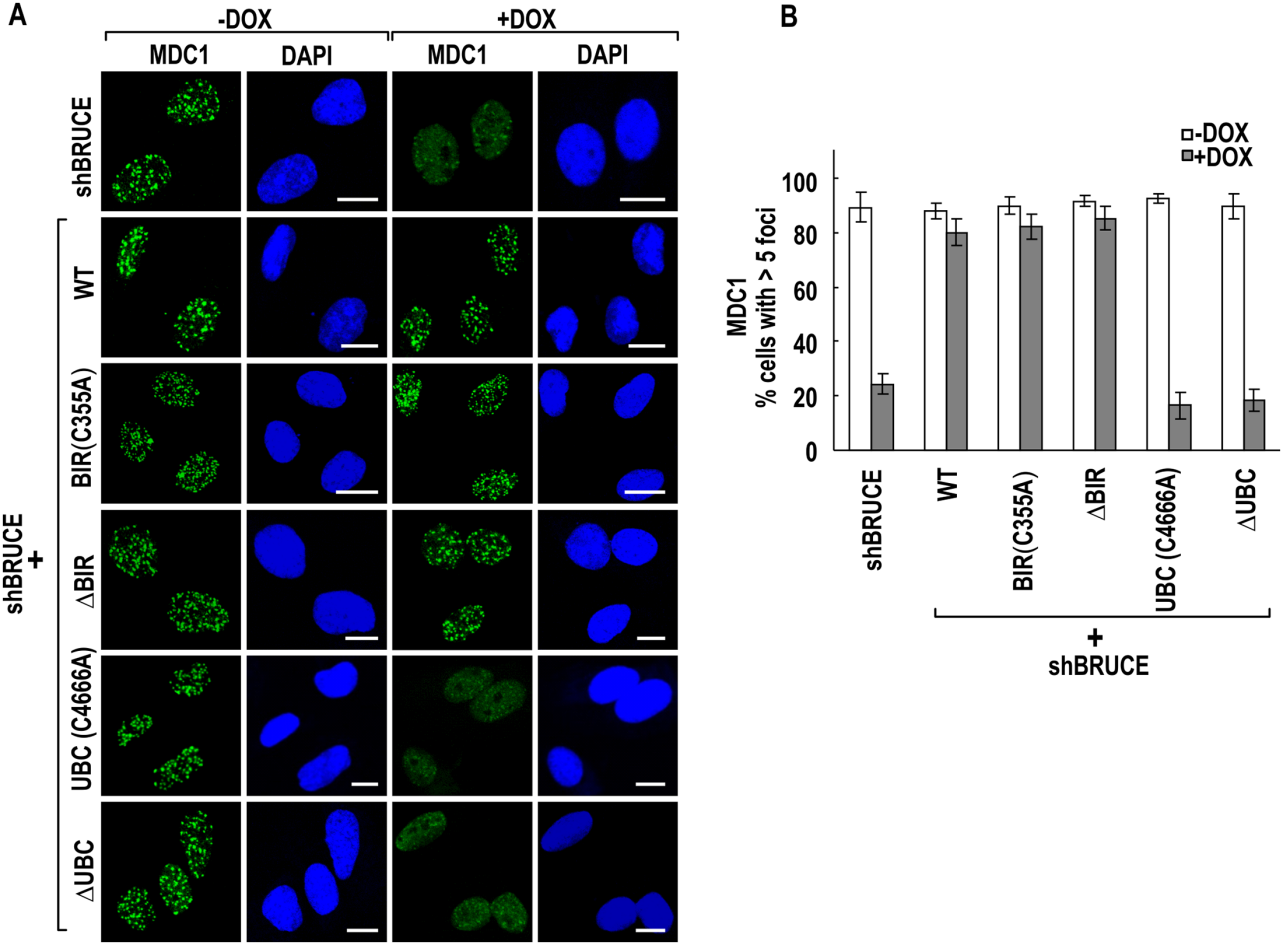
BRUCE UBC domain is required for MDC1 repair foci formation. U2OS-shBRUCE cells with stable expression of BRUCE or BRUCE mutants as indicated to the left were treated with DOX to knockdown endogenous BRUCE. After irradiation, cells were fixed and immunofluorescence stained for MDC1 (**A**) and quantified for cells with more than five nuclear foci (**B**). Bars, 10 μm. Error bars represent standard deviation from a triplicate of a representative experiment.

**Fig 6:**
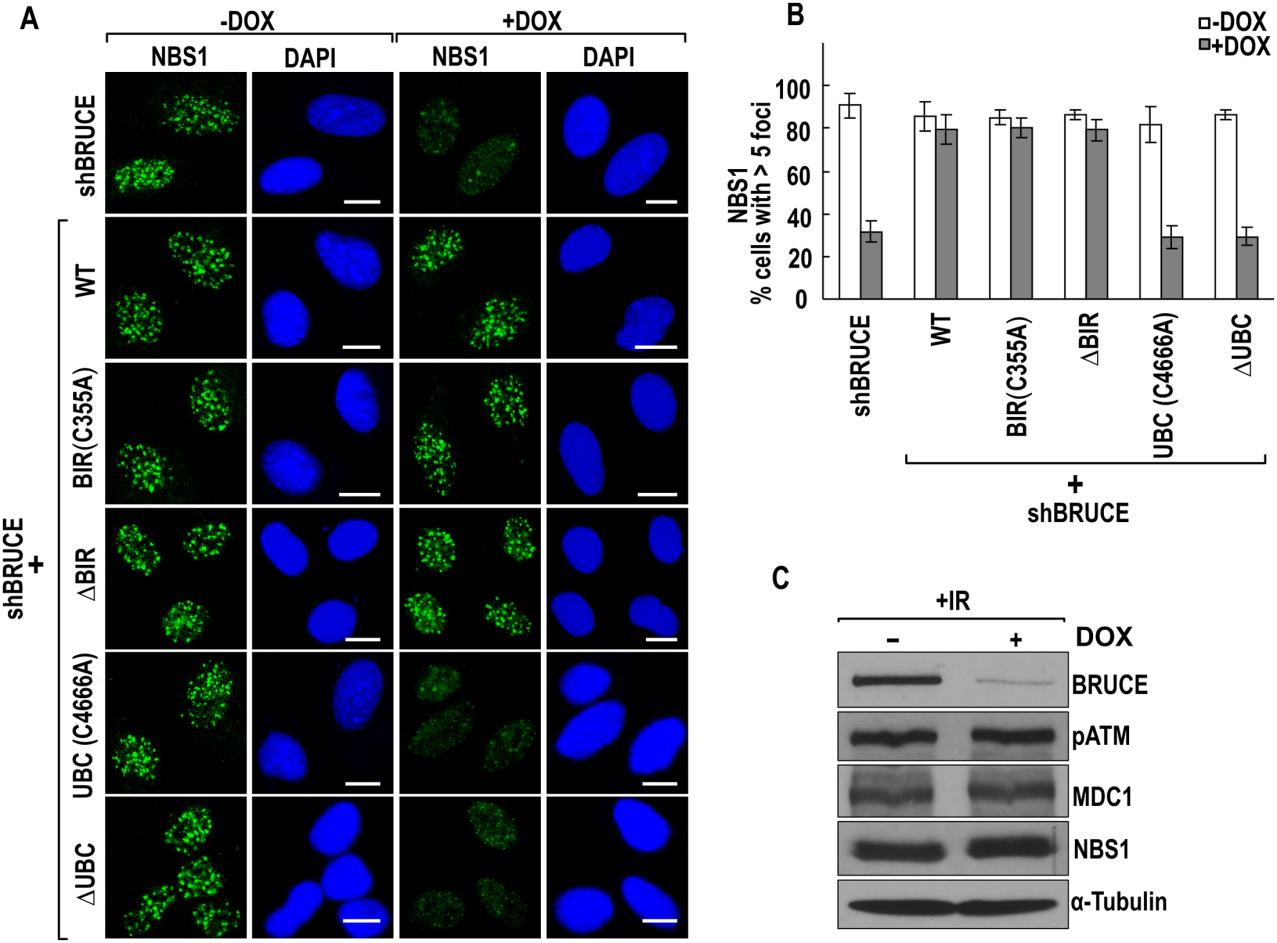
BRUCE UBC domain is required for NBS1 repair foci formation. U2OS-shBRUCE cells with stable expression of BRUCE or BRUCE mutants as indicated to the left were treated with DOX to knockdown endogenous BRUCE. After irradiation, cells were fixed and immunofluorescence stained for NBS1 (**A**) and quantified for cells with more than five nuclear foci (**B**). Bars, 10 μm. Error bars represent standard deviation from a triplicate of a representative experiment. DOX treated shBRUCE-U2OS cells were irradiated and the cell lysates subject to immunoblotting with the indicated antibodies showing the protein levels of ATM, MDC1 and NBS1 are not reduced post BRUCE knockdown (**C**).

Following exposure to IR, relaxation of chromatin structures by BRTI1-SWI-SNF pathway facilitates the accumulation at DSB of multiple early DDR factors, including pATM, NBS1, and MDC1 [39,40]. BRUCE is required for DNA damage foci formed by the above mentioned factors through permitting BRIT1-SWI-SNF mediated chromatin relaxation [24]. We next investigated whether the UBC and BIR domains of BRUCE affect the above events. Immunofluorescence staining of pATM, MDC1, and NBS1 in the six BRUCE stable U2OS cell lines showed that all three proteins could form DNA damage foci in irradiated cells that express BIR C355A or ΔBIR mutant BRUCE, but not UBC C4666A or ΔUBC mutant BRUCE (Figs 4–6, respectively). The lack of foci formation is not due to reduced production of respective proteins as the protein levels of pATM, MDC1 and NBS1 did not change after knockdown of BRUCE (Fig 6C). Together, these results indicate that the UBC domain is critical for BRUCE to promote the access of these early DDR signaling factors to DSBs by allowing for BRIT1-SWI-SNF mediated relaxation of chromatin induced by DNA damage.

### Inactivation of BRUCE UBC activity impairs HR repair of DNA DSB

**Fig 7:**
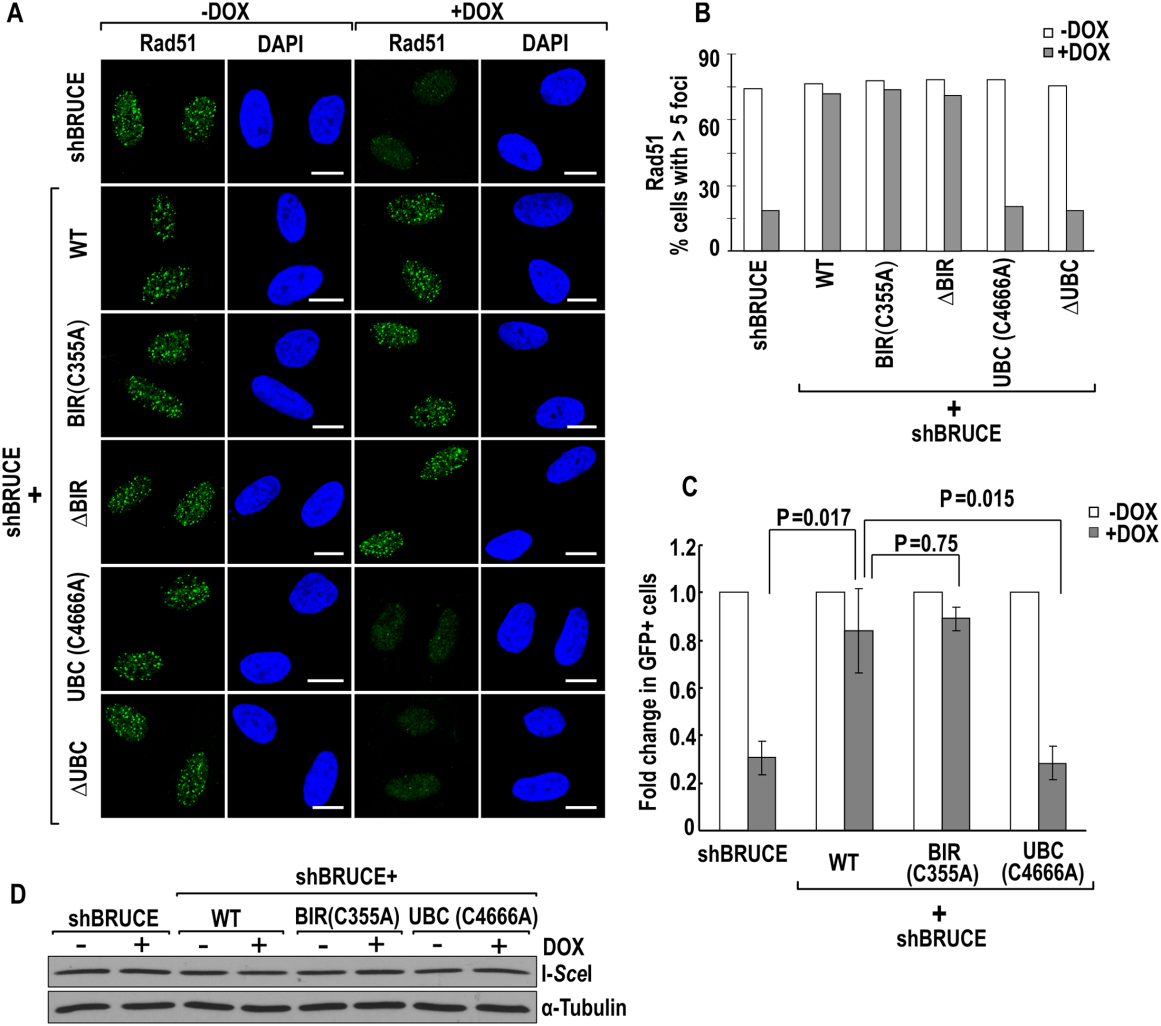
BRUCE UBC domain is required for RAD51 foci formation and HR repair. (**A**) BRUCE UBC domain is required for RAD51 foci formation. U2OS-shBRUCE cells with stable expression of BRUCE or BRUCE mutant as indicated to the left were treated with DOX to knock down endogenous BRUCE. Cells were irradiated, fixed and immunofluorescence stained for RAD51 (green) and counterstained with DAPI. Bars, 10 μm. (**B**) Quantification of RAD51 foci in (**A**). Cells with positive RAD51 foci (more than five foci) were quantitated and a total of more than two hundred cells were counted for each sample. (**C**) BRUCE UBC domain is needed for HR repair. U2OS stable cell lines as indicated were treated with DOX and transfected with the DR-GFP HR reporter and I-*Sce*I. Following 48 hrs of continued culture, cells were collected and subject to flow cytometry analysis. Percentage of GFP+ cells in control (no DOX treatment) were set as 1. Bars represent standard error of the mean (S.E.M) from four independent experiments. P value is calculated by Student’s *T*-test, two tailed. (**D**) Immunoblot showing expression level of HA-tagged I-*Sce*I in the samples presented in (**C**).

Recently we reported that knockdown of BRUCE resulted in a 50% reduction in homologous recombination (HR) repair but not much effect on NHEJ [24], two major pathways for DSB repair. Assembly of pATM, NSB1 and MDC1 at DSB-flanking chromatin is essential for DNA DSB repair. Since their assembly to DSB sites are impaired in BRUCE UBC-inactivated cells, we suspected that the UBC is required for HR. To examine this possibility, first we examined the repair foci formation by Rad51, a key HR recombinase that serves as a marker of HR [41–43]. Our results showed that Rad51 formed repair foci in cells with expression of wild type, BIR C355A, and ΔBIR mutant BRUCE but not UBC C4666A and ΔUBC (Fig 7A and 7B). Further, we assessed the requirement of UBC domain of BRUCE on HR repair of DSB induced by endonuclease I-*Sce*I that was established in Dr. Jasin Lab [44]. BRUCE stable U2OS cell lines of reconstituted WT, BIR C355A, or UBC C4666A mutant BRUCE were treated with DOX to induce shBRUCE-mediated depletion of endogenous BRUCE expression, followed by transfection with the DR-GFP reporter and an I-*Sec*I expression plasmid [37]. Subsequently, the percentage of GFP+ cells, indicative of HR efficiency, was compared. The UBC C4666A mutant BRUCE cell line shows 50% reduction in HR repair (Fig 7C), similar to the degree of reduction by depletion of endogenous BRUCE reported recently by our group [24]. The BIR C355A mutant BRUCE, on the other hand, retained the HR efficiency to the level comparable to wild type BRUCE (Fig 7C). Moreover, the reduced HR repair was not resulted from unequal transfection expression of the nuclease I-*Sce*I as the immunoblotting results showed an equal expression across the three cell lines (Fig 7D).

### The DNA repair defect in BRUCE-depleted cells is not a consequence of skewed cytokinesis

Prior to our elucidation of BRUCE in the regulation of DDR [24], it is known that BRUCE participates in cytokinesis as it is a midbody protein [28]. Although not quite likely but it remains as a reasonable concern that the observed impairment in DNA DSB signaling and repair could be a consequence of skewed cytokinesis. Therefore we examined this possibility in the following studies.

**Fig 8:**
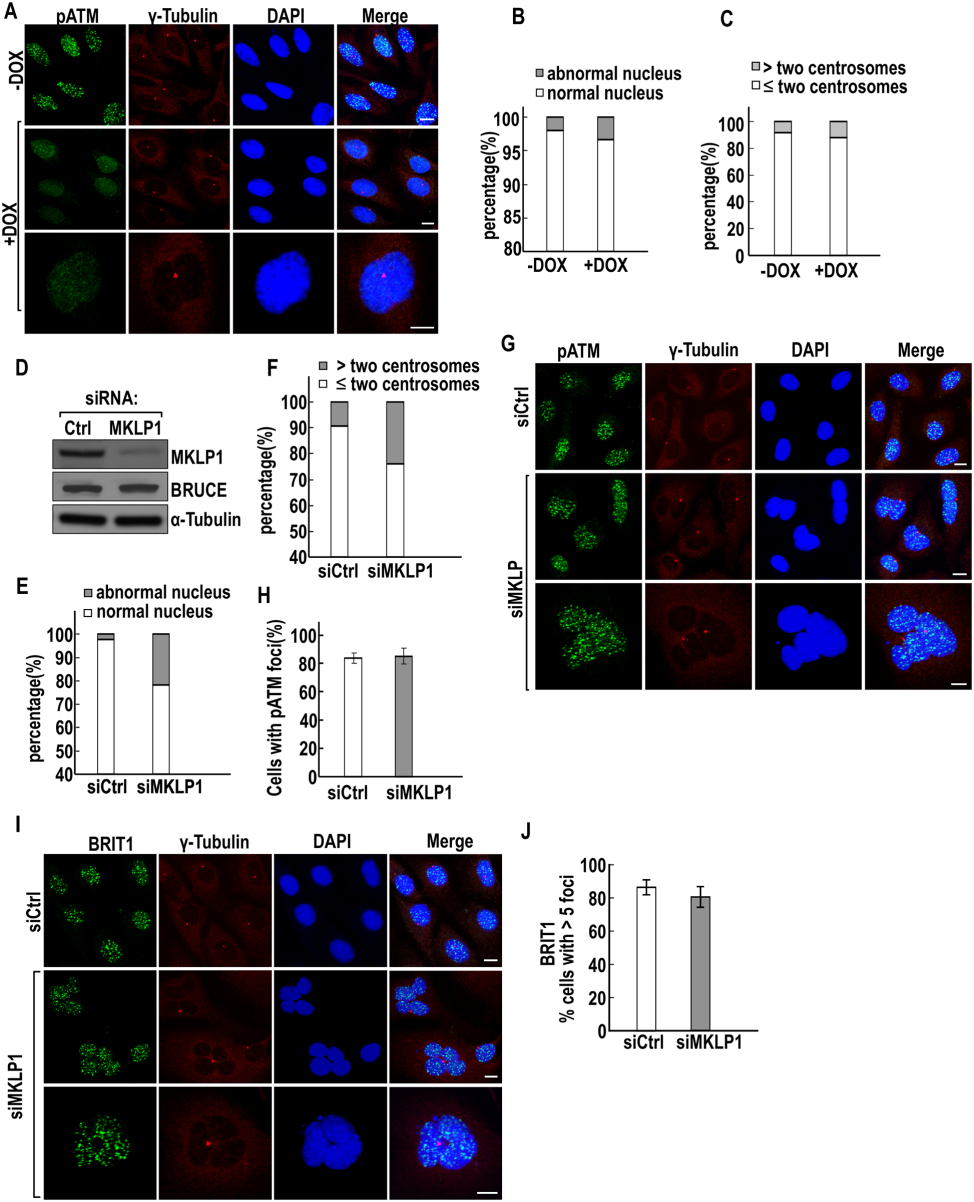
Impaired cytokinesis induced by MKLP1 depletion do not impair DNA repair. (**A**) Immunofluorescence staining of pATM (green) and centrosomes by γ-tubulin antibody (red) in U2OS-shBRUCE cells before and after DOX treatment with cell nucleus counterstained with DAPI. Bars, 10 μm. (**B**) and (**C**) Quantification of defective cytokinesis in irradiated shBRUCE-U2OS cells (5 Gy) with or without BRUCE depletion induced by Dox treatment, scored by counting bi- and multi-nucleated cells (**B**) and centrosome amplification by γ-tubulin staining (**C**). (**D**) Western blotting showing knockdown of MKLP1 by siRNA at 48 hrs post transfection of U2OS cells. (**E**) and (**F**) Quantification of cytokinesis defects in cells depleted of MKLP1 scored by bi- and multi-nucleated cells (**E**) and centrosome amplification (**F**). (**G**) and (**H**) immunofluorescence staining of pATM (green) and centrosomes (red) with cell nucleus counterstained with DAPI (blue) of shBRUCE-U2OS cells treated with siRNA targeting MKLP1 (**G**) and the pATM foci results were quantified in (**H**). Bars, 10 μm. Error bars represent standard deviation from a triplicate of a representative experiment. (**I**) and (**J**) immunofluorescence staining of BRIT1 (green) and centrosomes (red) with cell nucleus counterstained with DAPI (blue) of U2OS cells treated with the siRNA targeting MKLP1 (**I**) and the BRIT1 foci results were quantified in (**J**). Bars, 10 μm. Error bars represent standard deviation from a triplicate of a representative experiment.

The hallmarks of defective cytokinesis are bi- and multi-nucleated cells and amplification of centrosome numbers (more than 2 centrosomes/interphase cell) (29). It is noted that although BRUCE influences cytokinesis [28], the influence is not as significant as those essential cytokinesis factors such as MKLP1 [45,46]. Consequently, many cells with BRUCE depleted by siRNA go on to complete cell divisions normally [28]. In agreement with this notion, following BRUCE depletion by shRNA, the majority of U2OS cells had progressed through cytokinesis normally and most of the daughter cells are single nucleates with one or two centrosomes (Fig 8A). By conducting a careful quantification, only a negligible increase in the percentage of defective cytokinesis was observed, scored by counting bi- and multi-nucleated cells (Fig 8B). In agreement, a negligible increase was observed in the same cell population for amplification of centrosomes (Fig 8C). Such a minor effect of BRUCE depletion on cytokinesis is unlikely accountable for the significant DNA repair defect in BRUCE-depleted cells as shown in a number of figures in this study. Nonetheless, we further examined whether impairment of cytokinesis has any effect on DSB response in U2OS cell by depleting the essential cytokinesis protein MKPL1. As expected, cells depleted of MKPL1 (Fig 8D) exhibited a profound negative effect on cytokinesis with more than 20% of the cell population with impaired cytokinesis compared with control, assessed by multi-nucleated cells (Fig 8E) and centrosome numbers (Fig 8F). However, IR-induced pATM repair foci were equally formed in cells treated with Ctrl or MKLP1 siRNAs (Fig 8G and 8H). Moreover, BRIT1 repair foci were also readily formed in the two type siRNAs treated cells (Fig 8I and 8J). Together, these data demonstrated that cytokinesis and DNA repair are two independent events in the face of BRUCE inactivation.

## Discussion

Our recent study reported a new “BRUCE-USP8-BRIT1” axis in the DSB response pathways, in which the scaffold BRUCE and the Dub USP8 work together to determine the sub-nuclear localization and repair function of the DSB response protein BRIT1 [24]. That study identified “deubiquitination of BRIT1” as a new regulatory step for targeting BRIT1 to DNA damage sites prior to the known step of its recruitment by γ-H2AX at DSB. Specifically, in unstimulated cells, the scaffold BRUCE sequesters ubiquitinated BRIT1 onto the nuclear BRUCE-USP8-BRIT1 platform. Following exposure to IR, USP8 promotes BRIT1 deubiquitination in BRUCE-dependent manner, leading to dissociation of BRIT1 from the platform and consequent recruitment of it to the DSB sites by binding to γ-H2AX. Absent expression of BRUCE disrupts the complex and renders USP8 unable to catalyze deubiquitination of BRIT1, thereby abolishing BRIT1 repair foci, leaving chromatin in a compact configuration, and consequently impeding further accumulation of other DDR factors to DSB-spanning chromatin. Eventually, HR repair of DSB is impaired and genome instability ensured [24]. In current study, we investigated another important aspect in the “BRUCE-USP8-BRIT1” axis of DSB signaling pathways—the involvement of BRUCE domains. Our results support the notion that it is the UBC, not the BIR domain of BRUCE that is critical for BRIT1 deubiquitination, its recruitment to DNA damage sites and HR repair. Our data also clarify that the function of BRUCE in DSB signaling is separated from its roles in pro-cytokinesis and anti-apoptosis.

Ubiquitination plays an important role in the regulation of various cellular processes including DNA damage signaling and repair. A lot of work has elucidated how ubiquitination regulates the recruitment of DNA repair proteins, among which the most extensively studied is ubiquitination and deubiquitination of histones flanking DSB. For instance, H2A/H2AX ubiquitinated by the E3 ligase RNF8 and RNF168 serves as the landmarks for recruitment of key downstream repair factors 53BP1 and BRAC1 [6,7,9,10,12]. Since BRUCE UBC has a chimeric E2/E3 ligase activity [27], at first we suspected that BRUCE is the E3 ligase for BRIT1 ubiquitination and that such ubiquitinated BRIT1 is sequestered onto the BRUCE-USP8 platform in unstimulated cells as we recently reported [24]. However, our experimental results indicate the opposite—inactivation of BRUCE UBC or knockdown of BRUCE results in a significant increase in the ubiquitinated BRIT1 products. Therefore, BRUCE is not the E3 ligase that directly ubiquitinates BRIT1. Although the protein substrates of BRUCE E2/E3 activities in DDR have not been identified, we provide evident showing that, in contrast to the extensively studied E3 ligases RNF8 and RNF168 that mainly catalyze ubiquitination of H2A type of histones at damaged chromatin, the E3 ligase of BRUCE appears to regulate BRIT1 function, a step further upstream of histone modification and even regulates accumulation of pATM, NBS1 and MDC1 at DSB. These findings suggest that BRUCE function is distinct from RNF8 and RNF168. Therefore, this study provides the insight into further investigation of how BRUCE E2/E3 activities regulate DNA damage response.

**Fig 9:**
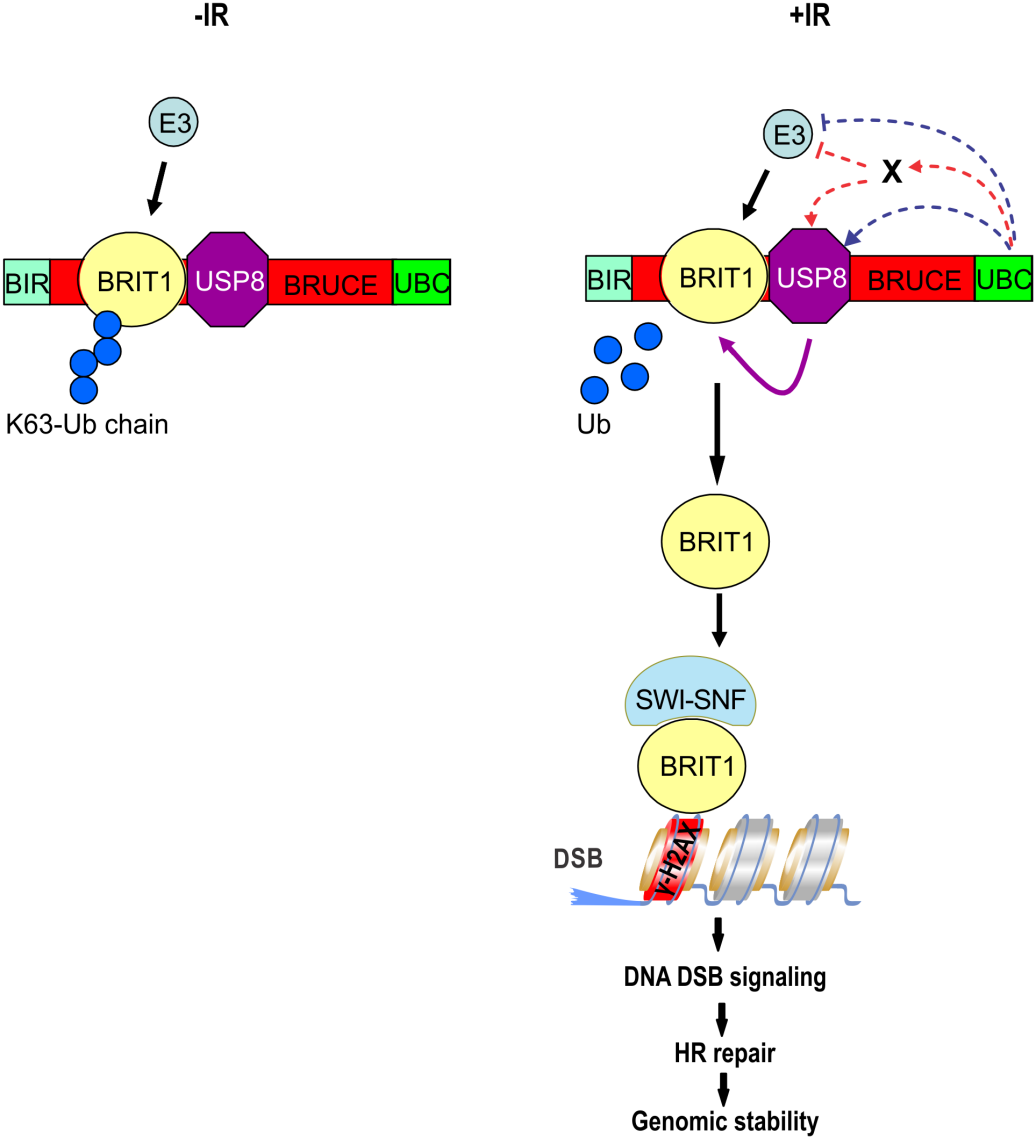
Working model for activation of BRIT1 deubiquitination by BRUCE UBC. Following exposure to IR, the E2/E3 activity of BRUCE increases and catalyzes ubiquitination of its substrate(s), yet to be identified, towards deubiquitination of BRIT1 and enhancement of DNA repair. The putative substrate of BRUCE could be USP8 or the E3 ligase for BRIT1 (blue lines), or another unknown protein X (red lines). In any case, assertion of BRUCE E2/E3 activity is expected to tip the balance towards deubiquitination of BRIT1 by promoting USP8 Dub function or/and by inhibiting the E3 ligase activity, thereby enhancing deubiquitination of BRTI1 and promoting DNA damage signaling and repair (see text for details).

Protein ubiquitination and deubiquitination are dynamic processes implicated in the regulation of numerous cellular pathways including DNA damage response. Thus, ubiquitination and deubiquitination of BRIT1 is likely also a dynamic process regulated by its E3 ligase (to be identified) and its Dub USP8, respectively. Based on this study and our previous report [24], we propose a working model for how BRUCE UBC might assert its role in DDR (Fig 9). In unstressed cells, as showed by our previous study, BRIT1 is K63-ubiquitinated in the region of 566-655aa [24]. Following DNA damage induction by exposure to IR, the E2/E3 activity of BRUCE increases and catalyzes ubiquitination of its substrate (s) yet to be identified. The putative substrate of BRUCE could be USP8 and if this is the case, ubiquitination of USP8 by BRUCE is expected to enhance its Dub activity over Ub-BRIT1, thereby facilitating removal of the Ub chains from K63-Ub-BRIT1 and promoting recruitment of de-ubiquitinated BRIT1 to sites of DNA damage. Alternatively, the putative substrate of BRUCE could be the E3 ligase of BRIT1 and if this is true, ubiquitination of that E3 ligase is expected to be inhibitory and therefore BRIT1 ubiquitination is attenuated, tipping the balance of “ubiquitination and deubiquitination” towards deubiquitination of BRIT1 by USP8, thereby promoting BRIT1 recruitment to DSB. In addition, the putative substrate of BRUCE could be an unknown protein X and when protein X is ubiquitinated, it tips the balance towards deubiquitination of BRIT1 and enhances DNA damage signaling and repair. We will investigate these possibilities in our future studies.

BRUCE has two previously characterized functions. It is an IAP and inhibits apoptosis when overexpressed by using its BIR domain, an IAP signature domain, to bind and inhibit Smac and caspases [26,29–33]. BRUCE is also a scaffold in mediating the integrity of midbody for cytokinesis and knockdown of BRUCE impairs the formation of midbody ring due to abnormal ubiquitin translocation to the midbody ring [28]. In addition to these two known functions, the involvement of BRUCE in the regulation of DSB signaling was reported recently by our group [24]. Therefore, it is critical to distinguish whether the impaired DSB signaling and repair in BRUCE depleted cells is the outcome of skewed cytokinesis or loss of apoptotic inhibition. In this study, we provided structural and functional data that support a separation of DSB repair function from the other two. First, regarding whether the DNA repair defect could come from impaired cytokinesis, our data demonstrated that the repair defect is not the consequence of skewered cytokinesis as abrogated formation of DNA repair foci post BRUCE depletion is independent of aberrant cytokinesis. Specifically, BRIT1 and pATM repair foci were abolished in cells derived from either normal or impaired cytokinesis as long as BRUCE expression was depleted. Further, knockdown of BRUCE only results in a small increase in the portion of cells with cytokinesis defect, whereas the large population of cells still have normal morphology, single nucleus, and normal number of centrosomes, but they are not capable to form DNA repair foci by pATM or BRIT1, confirming that the malfunction in DNA repair due to BRUCE depletion is not resulted from defective cytokinesis. Moreover, MKLP1 is a component of central spindle complex and essential for cytokinesis. Knockdown of MKLP1 interrupts midbody ring formation and results in incompletion of cytokinesis. However, in MKLP1-depleted cells where cytokinesis is impaired, there is no obvious reduction in the DNA repair foci formed by BRIT1 or pATM. Therefore, cytokinesis aberrance does not affect accumulation of repair proteins to DSB. Second, in addition to cytokinesis, hyperactive apoptosis due to loss of IAP function in BURCE-depleted cells could indirectly give rise to impaired DNA repair. However, our data indicated that the BIR domain does not participate in DDR. Specifically, the BIR is dispensible for the scaffold function of BRUCE in tethering USP8 and BRIT1 forming the nuclear BRUCE-USP8-BRIT1 complex [24]. It is neither required for USP8 mediated BRIT1 deubiquitination nor the formation of IR induced BRIT1 repair foci. Together, these structural and functional analyses support the notion that the new function of BRUCE in DSB signaling is separated from its roles in pro-cytokinesis and anti-apoptosis. In other words, the impaired DNA damage signaling in BRUCE knockdown cells is not the outcome of skewed cytokinesis or enhanced apoptosis.

Overall, the present study identified that the UBC domain of BRUCE is required for BRIT1 deubiquitination, BRIT1 foci formation, chromatin relaxation and homologous recombination repair. DNA repair, cytokinesis, and anti-apoptosis are three independent functions of BRUCE. In the future it would be interesting to identify the substrate of BRUCE E2/E3 ligase and the E3 ligase of BRIT1, which will provide the molecular basis for elucidating how BRUCE E2/E3 functions in the regulation of BRIT1 ubiquitination and deubiquitination through cooperation with USP8.
